# Association between leuko-glycemic index and mortality in critically ill patients with non-traumatic subarachnoid hemorrhage: analysis of the MIMIC-IV database

**DOI:** 10.3389/fneur.2025.1537585

**Published:** 2025-05-13

**Authors:** Hui Shen, Wei Guo, Jin Zhang, Qing Mei, Aihua Liu, Jiachun Liu

**Affiliations:** ^1^Cerebrovascular Disease Department, Neurological Disease Center, Beijing Anzhen Hospital, Capital Medical University, Beijing, China; ^2^Department of Interventional Neuroradiology, Beijing Neurosurgical Institute, Beijing Tiantan Hospital, Capital Medical University, Beijing, China; ^3^Department of Interventional Neuroradiology, Sanbo Brain Hospital, Capital Medical University, Beijing, China; ^4^Department of Neurosurgery, Beijing Friendship Hospital, Capital Medical University, Beijing, China; ^5^Department of Neurology, Beijing Pinggu Hospital, Beijing, China; ^6^Department of Neurosurgery, Beijing Hospital, National Center of Gerontology, Institute of Geriatric Medicine, Chinese Academy of Medical Sciences, Beijing, China

**Keywords:** Leuko-glycemic index, white cell, non-traumatic subarachnoid hemorrhage, prognosis, MIMIC-IV database, glucose

## Abstract

**Background:**

Non-traumatic subarachnoid hemorrhage (NTSAH), primarily caused by intracranial aneurysm rupture, represents a significant global health challenge due to its high mortality and morbidity. The leuko-glycemic index (LGI), a biomarker reflecting oxidative stress and inflammation, has been associated with adverse cardiovascular outcomes. However, its prognostic value in critically ill NTSAH patients remains uncertain. Understanding the relationship between LGI and patient outcomes is essential to improve clinical management of NTSAH.

**Methods:**

We identified NTSAH patients from the Medical Information Mart for Intensive Care-IV (MIMIC-IV, version 2.2) database. Participants were divided into quartiles based on their LGI scores. Mortality was evaluated at multiple time points: ICU stay, in-hospital, and at 1-, 6-, and 12-month post-admission. The association between LGI and mortality was examined using multivariate Cox proportional hazards regression. Restricted cubic spline (RCS) analysis was employed to delineate the relationship between LGI scores and mortality risk and to identify the cutoff value. The robustness of these findings were confirmed through subgroup analyses, interaction tests, and likelihood ratio tests.

**Results:**

A total of 750 patients were included, with 57% being female. Mortality rates were 17% in the ICU, 20% in-hospital, 21% at 1 month, 27% at 6 months, and 29% at 1 year. Multivariate Cox regression analysis revealed that higher LGI score were significantly associated with increased mortality at 1 month, 6 months, and 1 year. RCS analysis demonstrated a positive correlation between elevated LGI scores and mortality risk.

**Conclusion:**

LGI is significantly associated with mortality in critically ill NTSAH patients, suggesting its potential as a prognostic biomarker for risk stratification. Further validation through prospective cohort studies is necessary to confirm these findings.

## Introduction

Subarachnoid hemorrhage (SAH) is a devastating subtype of stroke characterized by bleeding into the subarachnoid space, usually caused by the rupture of blood vessels at the base or surface of the brain, leading to distinct clinical manifestations. Globally, SAH affects approximately 9.1 individuals per 100,000 annually (1), with intracranial aneurysm ruptures accounting for 5–10% of all stroke cases (2). The prognosis for SAH remains poor, with a case fatality rate of approximately 35% (3). Optimal management of non-traumatic SAH (NTSAH) requires prompt attention to early brain injury, delayed cerebral ischemia (DCI), increased intracranial pressure, and systemic complications. Timely, aggressive, and targeted therapeutic strategies are essential for improving clinical outcomes in SAH patients. Given the increasing incidence of NTSAH in intensive care units, identifying robust prognostic indicators that predict poor outcomes is crucial. These indicators should be simple, cost-effective, and easily integrated into clinical practice to enhance patient management and improve survival rates.

Identifying prognostic factors is essential for predicting adverse outcomes in critically ill patients with NTSAH. The Leuko-Glycemic Index (LGI), a relatively recent marker of inflammation, has been associated with poor outcomes in various conditions, including acute myocardial infarction (MI) (4), coronary artery disease (CAD) (5), COVID-19 (6), and arteriovenous fistula (AVF) failure in long-term dialysis patients (7). Acute hyperglycemia is widely recognized for its pro-inflammatory effects, and numerous studies, particularly those involving trauma models, have shown that glucose administration significantly enhances leukocyte adhesion and migration, indicating a close association between LGI and the intensity and nature of inflammatory responses (8). Research further suggests that NTSAH can trigger a complex inflammatory response, potentially worsening brain injury (9). However, the prognostic significance of LGI in critically ill patients with NTSAH remains uncertain, highlighting the need for additional research to establish its value as a prognostic biomarker.

In this study, we aimed to evaluate the prognostic significance of LGI as a mortality predictor in critically ill patients with NTSAH. We specifically investigated the correlation between LGI levels and mortality within this stroke subgroup, utilizing data from the Medical Information Mart for Intensive Care (MIMIC)-IV database. The objective of this research is to determine the predictive efficacy of LGI, with the goal of refining management strategies for NTSAH patients and facilitating the implementation of timely interventions for those at increased risk of mortality.

## Materials and methods

### Data source

This retrospective cohort study utilized data from the MIMIC-IV database (version 2.2). Data extraction was performed systematically using Navicat Premium (version 15). The MIMIC-IV database contains de-identified electronic health records of patients admitted to the Beth Israel Deaconess Medical Center from 2008 to 2019. The principal investigator, Hui Shen, completed the “Protecting Human Research Participants” online course (ID: 1741944) to gain access to the MIMIC-IV database. This manuscript was prepared in accordance with the Strengthening the Reporting of Observational Studies in Epidemiology (STROBE) guidelines. All personal identifiers were anonymized by replacing patient data with randomly assigned numbers. Given the anonymized nature of the data, neither ethical approval nor informed consent was required.

### Criteria for population selection

**Figure 1:**
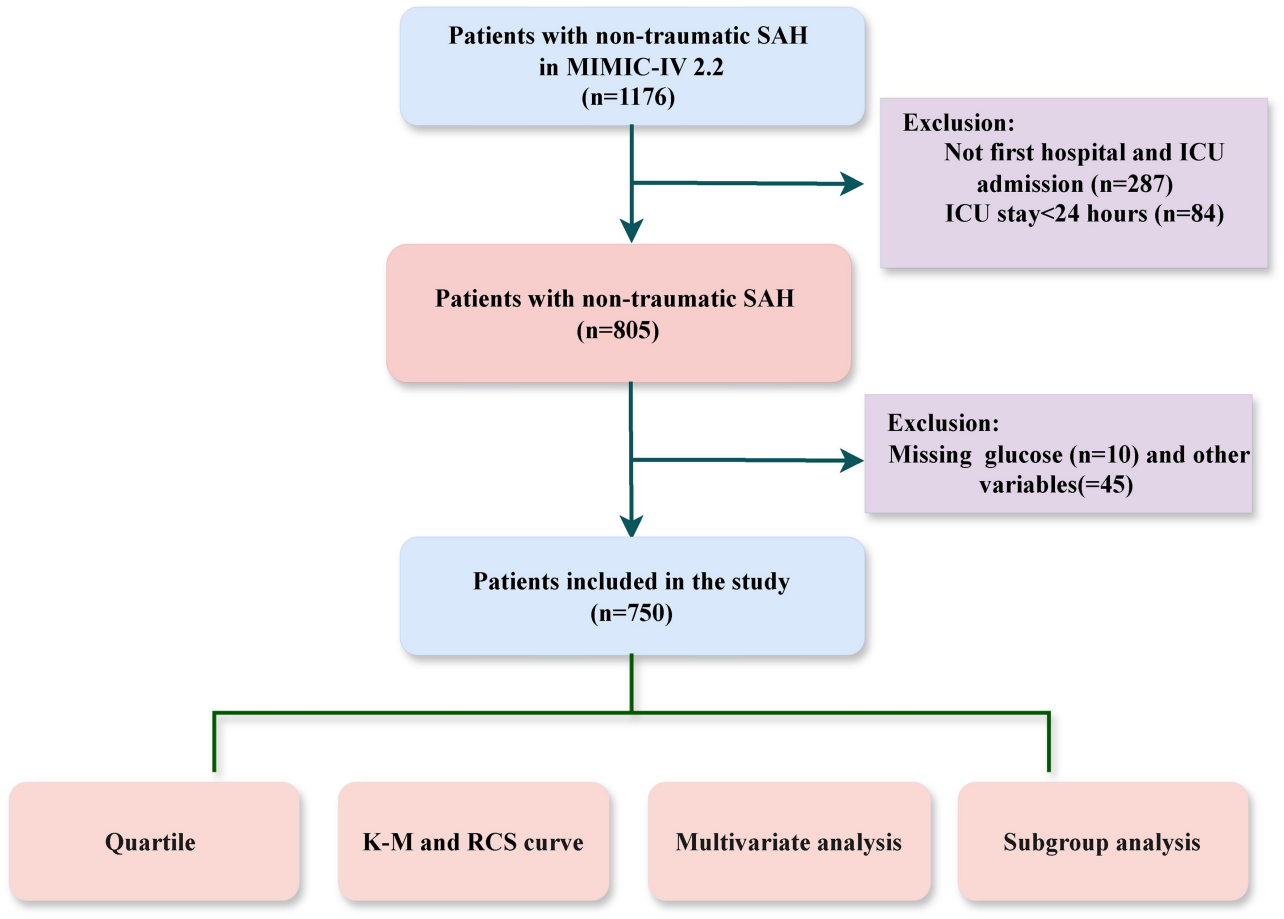
The flowchart of the cohort selection process.

The study cohort consisted of adult ICU patients diagnosed with NTSAH using ICD-9 code 430 and ICD-10 codes I60, identified from the MIMIC-IV database. Patients were excluded if they met any of the following criteria: (1) ICU stays shorter than 24 h; (2) non-first hospital or ICU admissions; (3) incomplete data, such as missing white blood cell counts or blood glucose levels. A total of 750 patients were included in the study cohort (Figure 1).

### Data collection

Baseline characteristics were extracted from the MIMIC-IV database within the first 24 h of ICU admission, covering five main domains: demographic data, clinical severity scores, mean vital signs and laboratory parameters, comorbidities, and ICU treatments. Laboratory parameters, including white blood cell count (WBC, ×10^9/L) and blood glucose levels (mmol/L), were measured from peripheral venous blood samples collected. Unlike the definitions used in previous studies (4–6), to better align with clinical applications. The LGI index was calculated using the formula “white blood cell count (WBC, ×10^9/L) × blood glucose levels (mmol/L).” Participants were then stratified into four quartiles (Q1, Q2, Q3, Q4) based on their LGI values, with Q1 serving as the reference group.

### Clinical outcomes

The primary outcome of this study was 6-month mortality, with 1-month and 1-year mortality serving as secondary outcomes. Deaths were documented based on their occurrence within the specified time frames following ICU admission, rather than merely noting whether the patient had died by a particular milestone. A comprehensive list of the extracted variables and outcomes is provided in Supplementary Table 1.

### Statistical analysis

Continuous variables were presented as medians with interquartile ranges (IQR), while categorical variables were shown as counts and percentages. Baseline characteristics among LGI quartile groups were compared using Pearson’s chi-square tests or Fisher’s exact tests for categorical variables and Kruskal-Wallis tests for continuous variables, as appropriate.

Kaplan–Meier survival analysis was used to compare endpoint incidence rates among the four LGI-stratified groups. Subsequently, multivariate Cox proportional hazards regression models were constructed to adjust for potential confounding factors in two models. Model 1 adjusted for age, race, and gender. Model 2 further included Glasgow Coma Scale (GCS) score, MI, and the administration of statins, nimodipine, coiling, and mechanical ventilation (MV).

LGI was analyzed both as a continuous variable (per 10-unit increase) and a categorical variable, with the first quartile (Q1) as the reference group. Trend analyses across quartiles were performed to determine *p*-values. The optimal LGI cutoff value associated with 6-month mortality was identified using the maximally selected rank statistics method with the “survminer” package. The relationship between LGI and hazard ratios (HRs) was explored using restricted cubic spline (RCS) regression with 4 knots, referencing the identified cutoff value, with the “plotRCS” package.

Stratification and interaction analyses were conducted to evaluate the impact of variables such as gender, age (<60 and ≥60 years), race, GCS (3–8 and 9–15), MI, diabetes mellitus (DM), sepsis, statin use, coiling and MV, with likelihood ratio tests used to investigate interactions. All statistical analyses were performed using R software, version 4.4.0, with a two-sided *p*-value of less than 0.05 considered statistically significant.

## Results

From the MIMIC-IV database, a cohort of 750 patients with NTSAH was identified. The majority of these patients, 439 (58.5%), were White, with a median age of 60.7 years (IQR: 51–72). Mortality rates at various follow-up points were as follows: ICU, 17%; in-hospital, 20%; 1-month, 21%; 6-month, 27%; and 1-year, 29%.

### Baseline characteristics

Baseline characteristics of critically ill patients with NTSAH, stratified by LGI quartiles, were shown in the Table 1. Patients were divided into four quartiles based on LGI levels at hospital admission: Q1 (8.3–73.8), Q2 (73.9–111.9), Q3 (112–116.2), and Q4 (116.3–952.2). The median LGI values for each quartile were as follows: Q1, 55.5 (IQR: 44.8–66.2); Q2, 92.1 (IQR: 82.6–99.8); Q3, 135.2 (IQR: 124.5–147.6); and Q4, 241.1 (IQR: 190.8–308.3).

**Table 1:** Characteristics and outcomes of participants categorized by leuko-glycemic index.

Characteristic	Overall *N* = 750^1^	Q1 (8.3–73.8) *N* = 188^1^	Q2 (73.9–111.9) *N* = 187^1^	Q3 (112–116.2) *N* = 188^1^	Q4 (116.3–952.2) *N* = 187^1^	p-value^2^
LGI	111.9 (73.8, 166.2)	55.5 (44.8, 66.2)	92.1 (82.6, 99.8)	135.2 (124.5, 147.6)	241.1 (190.8, 308.3)	<0.001
Demographic						
Female, *n* (%)	431 (57%)	112 (60%)	111 (59%)	99 (53%)	109 (58%)	0.5
Age, years	60.7 (51.0, 72.0)	60.8 (51.0, 73.4)	61.3 (52.4, 71.6)	57.8 (49.8, 72.5)	60.7 (52.0, 70.9)	0.7
White, *n* (%)	439 (59%)	117 (62%)	114 (61%)	114 (61%)	94 (50%)	0.068
Clinical severity						
APSIII	32.0 (24.0, 45.0)	29.0 (22.0, 38.0)	28.0 (22.0, 38.0)	32.5 (24.0, 45.0)	41.0 (30.0, 61.0)	<0.001
LODS	2.0 (1.0, 5.0)	2.0 (1.0, 4.0)	2.0 (1.0, 4.0)	3.0 (2.0, 5.0)	4.0 (2.0, 7.0)	<0.001
OASIS	29.0 (24.0, 37.0)	26.0 (22.5, 32.0)	27.0 (22.0, 33.0)	31.0 (25.0, 37.0)	34.0 (28.0, 40.0)	<0.001
GCS	14.0 (12.0, 15.0)	14.0 (13.0, 15.0)	14.0 (13.0, 15.0)	14.0 (10.0, 15.0)	14.0 (9.0, 15.0)	0.6
SIRS	3.0 (2.0, 3.0)	2.0 (1.0, 2.0)	2.0 (2.0, 3.0)	3.0 (2.0, 3.0)	3.0 (3.0, 4.0)	<0.001
SOFA	2.0 (1.0, 4.0)	2.0 (1.0, 3.0)	2.0 (1.0, 3.0)	3.0 (1.0, 4.0)	4.0 (2.0, 7.0)	<0.001
Vital signs						
HR, beats/min	77.3 (70.2, 86.5)	73.1 (66.8, 79.9)	77.2 (70.8, 84.6)	76.8 (70.4, 86.5)	83.3 (74.5, 92.8)	<0.001
SBP, mmHg	125.3 (116.5, 133.0)	123.6 (114.9, 133.1)	126.3 (117.9, 132.8)	127.6 (118.7, 134.2)	124.3 (114.6, 131.7)	0.027
DBP, mmHg	63.6 (57.8, 69.4)	63.4 (58.3, 70.3)	64.0 (58.3, 69.4)	63.2 (57.3, 68.2)	64.2 (57.4, 70.5)	0.5
MBP, mmHg	81.9 (75.9, 87.9)	81.5 (74.5, 88.1)	82.5 (76.4, 88.1)	81.8 (76.3, 87.6)	81.8 (75.6, 87.9)	0.8
RR, beats/min	17.6 (16.0, 19.8)	16.9 (15.5, 18.8)	17.3 (15.7, 18.5)	17.7 (16.0, 19.9)	19.1 (17.0, 22.2)	<0.001
Temperature, ^°^C	37.0 (36.8, 37.3)	36.9 (36.7, 37.2)	37.0 (36.8, 37.2)	37.1 (36.8, 37.3)	37.1 (36.7, 37.4)	0.037
Spo2	97.6 (96.1, 98.9)	97.3 (96.0, 98.3)	97.2 (95.9, 98.8)	98.0 (96.7, 99.2)	98.1 (96.4, 99.2)	<0.001
Laboratory tests						
Hemoglobin, g/dL	13.2 (12.0, 14.4)	12.8 (11.3, 13.7)	13.1 (12.0, 14.3)	13.5 (12.4, 14.6)	13.5 (12.1, 14.8)	<0.001
Platelets, 10^9^/L	231.0 (188.0, 281.0)	211.5 (171.5, 252.5)	221.0 (184.0, 263.0)	242.0 (203.0, 284.0)	263.0 (200.0, 321.0)	<0.001
WBC, 10^9^/L	13.1 (10.0, 16.6)	8.5 (7.3, 9.9)	11.8 (10.7, 13.0)	15.4 (13.6, 16.7)	20.3 (16.2, 24.1)	<0.001
Anion gap, mmoL/L	16.0 (14.0, 18.0)	15.0 (13.0, 17.0)	16.0 (14.0, 18.0)	16.0 (15.0, 18.0)	18.0 (15.0, 20.0)	<0.001
Bicarbonate, mmoL/L	24.0 (22.0, 26.0)	24.0 (22.0, 26.0)	24.0 (23.0, 26.0)	24.0 (22.0, 26.0)	23.0 (21.0, 26.0)	<0.001
BUN, mg/dL	16.0 (12.0, 20.0)	14.0 (11.0, 18.0)	15.0 (11.0, 20.0)	15.5 (12.0, 20.0)	17.0 (14.0, 22.0)	<0.001
Calcium, mmol/L	8.7 (8.3, 9.1)	8.7 (8.3, 9.2)	8.7 (8.3, 9.1)	8.8 (8.4, 9.0)	8.8 (8.3, 9.3)	0.8
Chloride, mmol/L	107.0 (104.0, 110.0)	106.0 (104.0, 109.0)	106.0 (103.0, 108.0)	107.0 (104.0, 110.0)	109.0 (105.0, 113.0)	<0.001
Creatinine, mg/dL	0.9 (0.7, 1.0)	0.8 (0.7, 1.0)	0.8 (0.7, 1.0)	0.8 (0.7, 1.0)	0.9 (0.8, 1.2)	<0.001
Glucose, mmol/L	8.2 (7.0, 10.1)	6.4 (5.8, 7.2)	7.7 (7.0, 8.4)	8.9 (7.9, 9.8)	12.4 (9.9, 15.4)	<0.001
Sodium, mmol/L	141.0 (139.0, 144.0)	140.0 (139.0, 143.0)	140.0 (138.0, 143.0)	141.0 (139.0, 143.5)	142.0 (139.0, 145.0)	<0.001
Potassium, mmol/L	4.1 (3.8, 4.5)	4.0 (3.8, 4.3)	4.1 (3.9, 4.5)	4.0 (3.8, 4.4)	4.3 (4.0, 4.7)	<0.001
INR	1.1 (1.1, 1.2)	1.1 (1.0, 1.2)	1.1 (1.0, 1.2)	1.1 (1.1, 1.2)	1.2 (1.1, 1.3)	<0.001
PTT, s	28.6 (25.7, 32.1)	29.5 (26.7, 33.5)	28.1 (25.3, 31.1)	27.7 (24.9, 31.8)	28.6 (25.8, 33.1)	0.002
Comorbidities						
MI, *n* (%)	57 (7.6%)	8 (4.3%)	7 (3.7%)	15 (8.0%)	27 (14%)	<0.001
CHF, *n* (%)	56 (7.5%)	12 (6.4%)	14 (7.5%)	8 (4.3%)	22 (12%)	0.043
LD, *n* (%)	32 (4.3%)	8 (4.3%)	9 (4.8%)	7 (3.7%)	8 (4.3%)	>0.9
DM, *n* (%)	96 (13%)	14 (7.4%)	12 (6.4%)	21 (11%)	49 (26%)	<0.001
RD, *n* (%)	45 (6.0%)	12 (6.4%)	10 (5.3%)	9 (4.8%)	14 (7.5%)	0.7
MC, *n* (%)	29 (3.9%)	9 (4.8%)	5 (2.7%)	4 (2.1%)	11 (5.9%)	0.2
Sepsis, *n* (%)	370 (49%)	51 (27%)	78 (42%)	107 (57%)	134 (72%)	<0.001
CCI	3.0 (2.0, 5.0)	3.0 (2.0, 5.0)	3.0 (2.0, 5.0)	3.0 (2.0, 5.0)	4.0 (2.0, 6.0)	0.034
Treatment						
Statin, *n* (%)	194 (26%)	47 (25%)	52 (28%)	44 (23%)	51 (27%)	0.7
Nimodipine, *n* (%)	483 (64%)	108 (57%)	128 (68%)	129 (69%)	118 (63%)	0.075
Nicardipine, *n* (%)	327 (44%)	65 (35%)	80 (43%)	104 (55%)	78 (42%)	<0.001
Vasopressin, *n* (%)	73 (9.7%)	9 (4.8%)	16 (8.6%)	13 (6.9%)	35 (19%)	<0.001
Coiling, *n* (%)	268 (36%)	60 (32%)	61 (33%)	81 (43%)	66 (35%)	0.093
MV, *n* (%)	372 (50%)	48 (26%)	75 (40%)	107 (57%)	142 (76%)	<0.001
Outcomes						
ICU mortality	125 (17%)	13 (6.9%)	20 (11%)	30 (16%)	62 (33%)	<0.001
Hospital mortality	150 (20%)	17 (9.0%)	24 (13%)	38 (20%)	71 (38%)	<0.001
1-month mortality	160 (21%)	22 (12%)	23 (12%)	40 (21%)	75 (40%)	<0.001
6-month mortality	203 (27%)	35 (19%)	33 (18%)	50 (27%)	85 (45%)	<0.001
1-year mortality	214 (29%)	38 (20%)	37 (20%)	53 (28%)	86 (46%)	<0.001

^1^Median (Q1, Q3); *n* (%). ^2^Kruskal-Wallis rank sum test; Pearson’s Chi-squared test; Fisher’s exact test. LGI, leuko-glycemic index; SAPS III, Simplified Acute Physiology Score; LODS, logistic organ dysfunction system; OASIS, oxford acute severity of illness; GCS, Glasgow Coma Scale; SIRS systemic inflammatory response syndrome; SOFA, sequential organ failure assessment; HR, heart rate; SBP, systolic blood pressure; DBP, diastolic blood pressure; MBP, mean blood pressure; RR, respiratory rate; SpO2, percutaneous oxygen saturation; WBC, white blood cell; BUN, blood urea nitrogen; INR, international normalized rate; APTT; activated partial thromboplastin time; MI, myocardial infarction; CHF, chronic heart failure; LD, liver disease; DM, diabetes mellitus; RD, renal disease; MC, malignant cancer; CCI, Charlson comorbidity index; MV, mechanical ventilation.

Patients in the higher LGI quartiles exhibited more severe illness scores at admission, with the exception of the GCS score, and had a higher prevalence of comorbidities such as MI, chronic heart failure (CHF), DM, and sepsis. They also presented with higher Charlson Comorbidity Index (CCI) scores and elevated levels of laboratory parameters, including WBC, platelets, hemoglobin, creatinine, sodium, and international normalized ratio (INR). Furthermore, there was an increased use of ventilation, nicardipine, and vasopressor treatments, along with elevated mortality rates in the ICU, in-hospital, at 1-month, 6-month, and 1-year, compared to patients in the first quartile.

### Clinical outcomes

**Figure 2:**
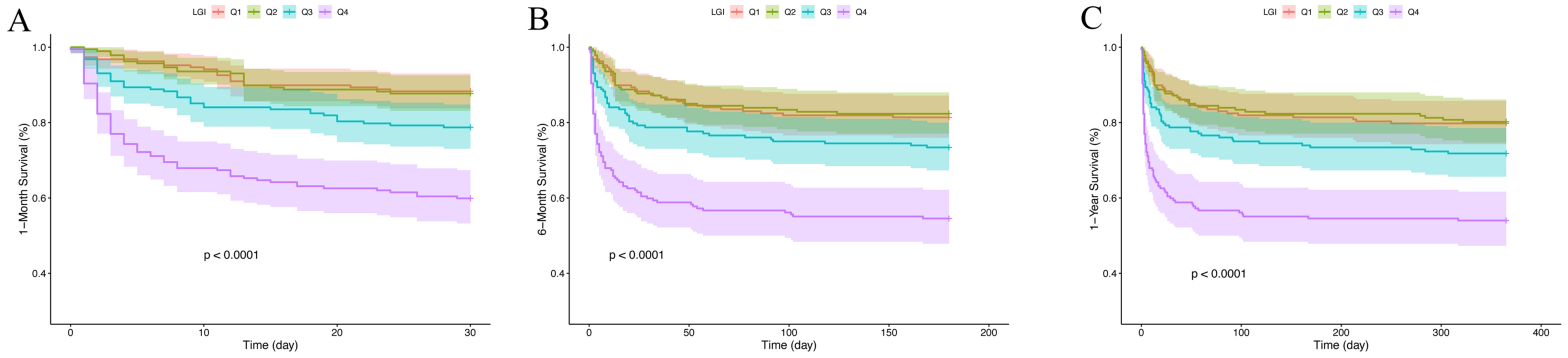
Kaplan–Meier survival analysis curves for all-cause mortality. LGI: Q1 (8.3–73.8), Q2 (73.9–111.9), Q3 (112.0–166.2), and Q4 (166.2–952.2). Kaplan–Meier curves for all-cause mortality according to groups at 1-month **(A)**, 6-month **(B)** and 1-year **(C)**.

Kaplan–Meier survival analysis was employed to evaluate the occurrence of different outcomes across LGI quartile groups, as depicted in Figure 2. The analysis revealed that patients with elevated LGI levels had a significantly increased risk of mortality at 1-month, 6-month, and 1-year intervals (*p* < 0.001).

A Cox proportional hazards model was then applied to evaluate the relationship between LGI and mortality at the specified intervals. The analysis confirmed LGI as a significant predictor of 1-month mortality in both Model 1 (HR: 1.04; 95% CI: 1.03–1.05; *p* < 0.001) and Model 2 (HR: 1.04; 95% CI: 1.03–1.05; *p* < 0.001) when treated as a continuous variable. When LGI was treated as a category variable, individuals in the fourth quartile (Q4) showed a significantly elevated risk of 1-month mortality in the Cox models: Model 1 (Q4 vs. Q1, HR: 4.35; 95% CI: 2.70–7.01; *p* < 0.001) and Model 2 (Q4 vs. Q1, HR: 2.42; 95% CI: 1.47–3.99; *p* < 0.001), indicating a rising trend in mortality risk with increasing LGI levels (P for trend < 0.001). This trend was consistent in the multivariate Cox analyses for 6-month and 1-year mortality rates. The detailed associations between LGI and mortality at 1-month, 6-month, and 1-year are outlined in Table 2.

**Table 2:** Cox proportional hazard models for 1-month and 6-month and 1-year all-cause mortality.

Outcome	Model 1			Model 2		
	HR (95% CI)	*p*-value	P for trend	HR (95% CI)	*p*-value	P for trend
1 month mortality						
Continuous variable per 10 unit	1.04 (1.03–1.05)	<0.001		1.03 (1.02–1.04)	<0.001	
Quartile			<0.001			<0.001
Q1 (*N* = 188)	Reference			Reference		
Q2 (*N* = 187)	1.05 (0.59–1.90)	0.9		0.81 (0.45–1.47)	0.5	
Q3 (*N* = 188)	2.10 (1.25–3.53)	0.005		1.45 (0.85–2.48)	0.2	
Q4 (*N* = 187)	4.35 (2.70–7.01)	<0.001		2.42 (1.47–3.99)	<0.001	
6-month mortality						
Continuous variable per 10 unit	1.04 (1.03–1.05)	<0.001		1.03 (1.03–1.04)	<0.001	
Quartile						<0.001
Q1 (*N* = 188)	Reference		<0.001	Reference		
Q2 (*N* = 187)	0.96 (0.59–1.54)	0.9		0.76 (0.47–1.23)	0.3	
Q3 (*N* = 188)	1.69(1.09–2.60)	0.018		1.16(0.74–1.81)	0.5	
Q4 (*N* = 187)	3.28 (2.21–4.87)	<0.01		1.93 (1.27–2.93)	0.002	
1-year mortality						
Continuous variable per 10 unit	1.04 (1.03–1.05)	<0.001		1.03 (1.02–1.04)	<0.001	
Quartile			<0.001			<0.001
Q1 (*N* = 188)	Reference			Reference		
Q2 (*N* = 187)	0.99 (0.63–1.55)	>0.9		0.80 (0.51–1.27)	0.3	
Q3 (*N* = 188)	1.66 (1.09–2.52)	0.018		1.16 (0.75–1.79)	0.5	
Q4 (*N* = 187)	3.08 (2.10–4.52)	<0.001		1.89(1.26–2.83)	0.002	

HR, hazard ratio; CI, confidence interval; Model 1: adjusted age, gender, and race; Model 2: adjusted age, gender, race, GCS, MI, statin, nimodipine, coiling, MV. GCS, Glasgow Coma Scale; MI, myocardial infarction.

**Figure 3:**
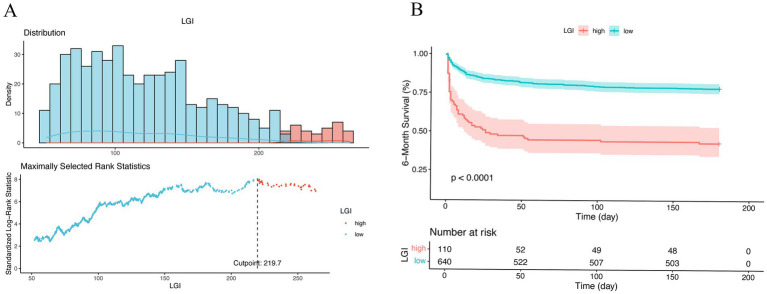
Optimal cutoff value for stratification of non-traumatic SAH patients into low- and high-risk groups were calculated by maximally selected rank statistics **(A)**. Kaplan–Meier survival analysis for all patients according to our risk stratification **(B)**. Survival curve showed the 6-month survival of the high-risk (red) and low-risk (blue) groups in the cohort. LGI, leuko-glycemic index.

**Figure 4:**
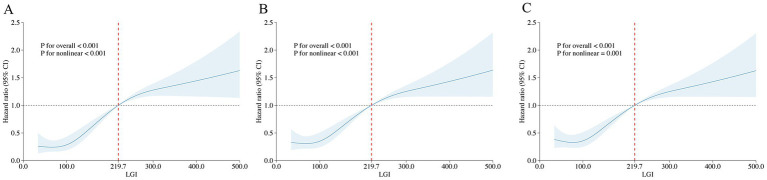
Restricted cubic spline analysis of LGI with all-cause mortality. Restricted cubic spline analysis of univariate model of LGI with 1-month **(A)**, 6-month **(B)**, and 1-year **(C)** all-cause mortality. LGI 219.7 was selected as the reference level represented by the vertical dotted lines.

The optimal cutoff value for the LGI of 219.7, identified using maximally selected rank statistics, was significantly associated with 6-month survival outcomes. Based on this threshold, participants were dichotomized into two groups: those with LGI ≤ 219.7 (*n* = 640) and those with LGI > 219.7 (*n* = 110), as illustrated in Figure 3. Furthermore, the application of RCS regression modeling revealed a nonlinear relationship between 1-month mortality risk and increasing LGI levels (P for non-linearity < 0.001). This nonlinear pattern was consistently observed across both the 6-month and 1-year mortality rates, as depicted in Figure 4.

### Subgroup analysis

**Figure 5:**
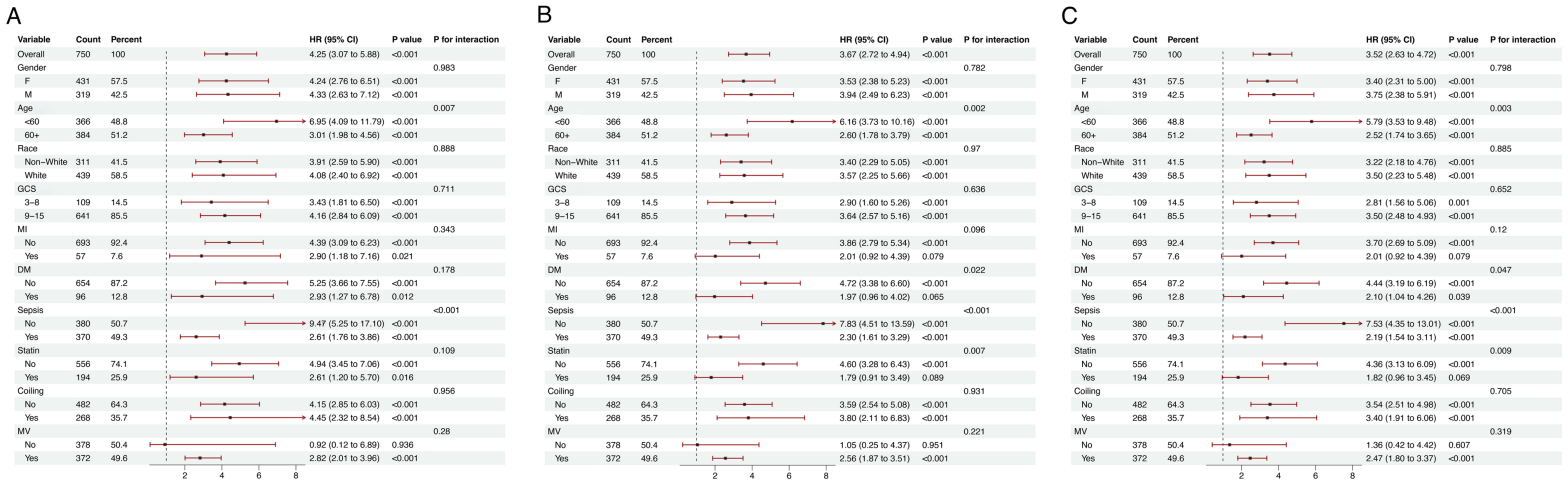
Forest plots illustrating stratified analyses of association of LGI and 1-month **(A)**, 6-month **(B)**, and 1-year **(C)** all-cause mortality. LGI, leuko-glycemic index; GCS, Glasgow Coma Scale; MI, myocardial infarction; DM, diabetes mellitus; MV, mechanical ventilation.

The prognostic significance of the LGI in predicting mortality was assessed across diverse demographic and clinical subgroups, including gender, age, ethnicity, GCS, MI, DM, sepsis, statin use, coiling and MV. Consistently, LGI was identified as a robust predictor of elevated 1-month mortality risk within each stratum. Similar trends were observed in analyses stratifying the association between LGI and both 6-month and 1-year mortality (Figure 5).

## Discussion

This study examined the correlation between the LGI and clinical outcomes in critically ill patients with NTSAH utilizing the MIMIC database. The results indicate that higher LGI levels are robustly associated with poor outcomes, manifesting both in the short and long-term prognosis. Even after controlling for confounding variables, LGI remained a significant predictor of outcomes over the study period. RCS regression analysis highlighted a nonlinear relationship between LGI and multiple outcomes. Thus, LGI may be a valuable prognostic indicator in clinical decision-making and should be recognized as an independent risk factor.

LGI has emerged as a potential biomarker for a spectrum of conditions, including COVID-19, preeclampsia, AVF failure in hemodialysis patients, and CAD (4). Wendy Marilú and colleagues have posited that LGI may be indicative of disease severity in COVID-19 patients (6). In a parallel vein, Adrian Vasileu’s research has highlighted that elevated preoperative LGI is significantly associated with the long-term failure of AVFs in dialysis patients, after adjusting for various confounding factors (7). Moreover, LGI has been recognized as a promising predictor of subsequent cardiovascular events, especially within the realm of CAD. Oguz Kilic’s study revealed that increased LGI levels are predictive of CAD severity in patients presenting with Canadian Cardiovascular Society (CCS) class 1 angina, and these levels show a correlation with the Gensini score (5). In addition, a meta-analysis has confirmed the prognostic value of LGI in forecasting in-hospital mortality rates subsequent to MI. Despite substantial evidence for LGI’s role in cardiovascular prognosis, a thorough systematic review of its relevance to cerebrovascular diseases, especially NTSAH, is absent. Addressing this gap, our study delineated the correlation between LGI and clinical prognosis in patients with NTSAH. We confirmed a significant association between LGI and adverse clinical outcomes at 1 month, 6 months, and 1-year post-event. This underscores LGI’s potential as a predictive tool for clinical outcomes in both cerebrovascular and cardiovascular settings.

Although LGI holds clinical promise, its application has been under scrutiny due to potential confounding by hyperglycemia. Stress and inflammatory responses, pivotal in diseases such as ischemic stroke, DM, renal disease, and various autoimmune disorders, have been extensively researched (10). Notably, several studies, including systematic reviews, have indicated that non-diabetic patients are at a higher risk of short-term mortality following acute ischemic stroke than diabetic patients (11–13). Moreover, a study by Shoujiang You et al. found that the combined predictive value of WBC count and blood glucose levels for in-hospital mortality was superior to either factor alone, with no significant difference between diabetic and non-diabetic patients (14). These findings suggested that, despite hyperglycemia concerns, LGI could be a validated and reliable marker across a broad spectrum of cerebrovascular conditions.

The precise pathophysiological mechanisms linking LGI to the etiology and mortality of NTSAH are not fully elucidated. Oxidative stress and inflammatory responses are hypothesized to mediate this association. Research indicated that increased WBC counts are associated with the onset of DCI, a pivotal determinant of poor clinical outcomes (15–17). WBC are implicated in early brain injury by causing hypoperfusion and ischemia when they become trapped in capillaries, forming aggregates that impede microcirculation. Additionally, WBC release cytotoxic mediators, including interleukins, which enhance WBC-endothelial interactions, exacerbating vascular and tissue damage (18). An elevated WBC count, indicative of systemic inflammation, was associated with an increased risk of SAH (19). Furthermore, research has demonstrated that an increased WBC count is correlated with the incidence of cardiovascular events, such as arteriosclerosis, and stroke (20).

Oxidative stress is posited to be a central factor in the short- and long-term pathogenesis of NTSAH (21). The sequelae of oxidative stress post-SAH encompass neuroinflammation, blood–brain barrier (BBB) disruption, and the generation of spasmogens (22, 23). Additionally, a reciprocal relationship between oxidative stress-induced hyperglycemia and hyperglycemia-induced oxidative stress has been established (24). Stress-induced hyperglycemia (SIH) frequently occurs in SAH patients, with studies indicating that hyperglycemia not only stimulates the production of inflammatory mediators but also aggravates brain injury by augmenting oxidative stress and compromising neuronal survival (25). SIH is a significant, independent risk factor for symptomatic vasospasm, DCI, and poor outcomes in patients with NTSAH (26). Consequently, LGI is anticipated to function as a composite biomarker of oxidative stress and inflammation, shedding light on the pathophysiological mechanisms of SAH.

Several limitations warrant acknowledgment in this study. First, the retrospective nature of our research introduces potential for selection bias. Furthermore, despite employing multivariate adjustments and subgroup analyses, the possibility of residual confounding remains due to the omission of certain variables in our dataset, such as Hunt-Hess score and modified Fisher scale. Additionally, our investigation was confined to the assessment of baseline LGI, neglecting to track its temporal variations during hospitalization and ICU stays. Therefore, subsequent studies should aim to elucidate the prognostic implications of LGI changes over time and assess how these fluctuations could impact patient outcomes.

## Conclusion

In summary, LGI is significantly correlated with mortality in critically ill patients with NTSAH. This correlation highlights the potential utility of LGI as a prognostic biomarker for stratifying patients according to their mortality risk.

## Data Availability

The original contributions presented in the study are included in the article/Supplementary material, further inquiries can be directed to the corresponding authors.
