# Supplementary material for: Association between leuko-glycemic index and mortality in critically ill patients with non-traumatic subarachnoid hemorrhage: analysis of the MIMIC-IV database
Source: Front Neurol. 2025 May 13;16:1537585. doi: 10.3389/fneur.2025.1537585 (PMC12106016; doi:10.3389/fneur.2025.1537585)
Supplement: Supplementary file 1 [file Table_1.docx]

**Supplementary table 1** The variables and outcomes in the study.

| **Items** | **Variables** |
| --- | --- |
| Demographic | age, gender, race |
| Clinical severity | APSIII, LODS, OASIS, GCS, SOFA |
| Vital sign | HR, SBP, DBP, MBP, RR, temperature, SpO2 |
| Laboratory test | Hemoglobin, platelets, WBC, anion gap, bicarbonate, BUN, calcium, chloride, creatinine, glucose, sodium, potassium, INR, APTT, LGI |
| Comorbidity | MI,CHF, LD, DM, RD, MC, sepsis, CCI |
| Treatment | statin, nimodipine, nicardipine, vasopressin, coiling, MV |
| Outcome | ICU mortality, hospital mortality, 1-month mortality, 3-month mortality, 1-year mortality |

SAPS III, Simplified Acute Physiology Score; LODS, logistic organ dysfunction system; OASIS, oxford acute severity of illness; GCS, Glasgow Coma Scale; SIRS systemic inflammatory response syndrome; SOFA, sequential organ failure assessment; HR, heart rate; SBP, systolic blood pressure; DBP, diastolic blood pressure; MBP, mean blood pressure; RR, respiratory rate; SpO2, percutaneous oxygen saturation; WBC, white blood cell; BUN, blood urea nitrogen; INR, international normalized rate; APTT; activated partial thromboplastin time; LGI, leuko-glycemic index; MI, myocardial infarction; CHF, chronic heart failure; LD, liver disease; DM, diabetes mellitus; RD, renal disease; MC, malignant cancer; CCI, Charlson comorbidity index,MV,mechanical ventilation
